# Diagnostic and therapeutic approaches to a case of pregnancy complicated by bilateral adrenocortical adenomas with primary aldosteronism and Cushing’s syndrome

**DOI:** 10.1007/s12020-024-04058-x

**Published:** 2024-10-08

**Authors:** Yanxi Chen, Lu Tan, Tao Chen, Haoming Tian, Li Li, Yan Ren

**Affiliations:** 1 Department of Endocrinology and Metabolism, Adrenal center, West China Hospital, Sichuan University, Chengdu, China; 2 Institute of Clinical Pathology, West China Hospital, Sichuan University, Chengdu, China

**Keywords:** Pregnancy, Bilateral adrenal adenomas, Primary aldosteronism, Cushing’s syndrome

## Abstract

Aldosterone/cortisol co-secreting adenomas (A/CPA) are a rare type of primary aldosteronism(PA), and cases of aldosterone/cortisol co-secreting adenomas during pregnancy are extremely rare, with no reported cases to date. The unique physiological state of pregnancy increases cortisol secretion through the hypothalamic-pituitary-adrenal (HPA) axis and leads to elevated levels of all components of the renin-angiotensin-aldosterone system (RAAS). This can cause overlapping symptoms with abnormal cortisol and aldosterone secretion, making diagnosis very challenging. This case involves a 29-year-old woman who developed hypercortisolism at 33 weeks of pregnancy. Despite receiving treatment for her symptoms and having a successful delivery, she continued to experience hypertension and hypokalaemia after giving birth. Eventually, she was diagnosed with ACTH-independent Cushing’s syndrome and primary aldosteronism due to independent cortisol and aldosterone secretion from bilateral adrenal adenomas. Following a thorough diagnosis, classification, treatment, and follow-up, the patient achieved a clinical cure while preserving normal adrenal function. Further investigation revealed that both diseases were caused by KCNJ5 and PRKACA mutations found in the bilateral adrenal adenomas.

## Case presentation

A 29-year-old woman at 33 weeks of gestation presented to a local hospital with persistent hypertension. She experienced severe headaches, and her blood pressure fluctuated between 130–170/90–110 mmHg, with a maximum systolic pressure of 180 mmHg. The patient had a history of irregular menstruation, with cycles of 1–3 months and periods lasting 10 days. She was G3P1, with a pregnancy termination at more than 6 months and one missed miscarriage. No abnormalities were observed before this pregnancy. At 25 weeks of gestation, she was diagnosed with gestational diabetes and developed oedema in both lower limbs without apparent cause. At 31 weeks, routine prenatal examination revealed normal systolic blood pressure but elevated diastolic pressure (the details were unclear), and her blood pressure continued to rise thereafter. Upon admission, physical examination revealed a rounded face; thin skin; acne on the face, neck, chest, and back; limb thinning, abdominal distension; and generalized weakness. She had no fever, chills, chest tightness, nausea, or vomiting. Laboratory tests revealed concurrent hypothyroidism (free thyroxine 0.42 ng/dL, thyroid stimulating hormone 0.514 mIU/L) and hypokalaemia (2.64 mmol/L). The patient was given symptomatic treatment, including promoting fetal lung maturation, antihypertensive and antispasmodic therapy, and potassium supplementation. Upon re-examination, her blood potassium level was 3.59 mmol/L. After communication with the patient and her family, an emergency caesarean section was performed five days later.

**Fig. 1:**
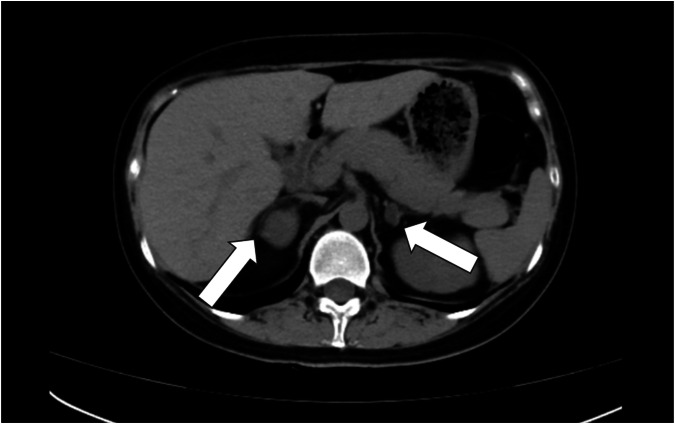
CT imaging of the adrenal glands shows masses on both sides. ) Right adrenal mass, with a slightly hypodense lesion measuring 3.3 × 2.2 cm in its largest cross-section; ) left adrenal mass, with a slightly hypodense lesion measuring 1.3 × 0.8 cm in its largest cross-section

On the 50th postpartum day, the patient’s hypertension and hypokalaemia persisted. An abdominal computed tomography (CT) scan revealed bilateral adrenal masses, the largest on the right measuring 3.3 × 2.2 cm and that on the left measuring 1.3 × 0.8 cm (Fig. 1). Laboratory assessment revealed significantly elevated morning cortisol concentrations (858.00 nmol/L; reference range 185–624 nmol/l) and suppressed adrenocorticotropin (ACTH) level were <1.00 ng/L(reference range 5–78 ng/L), whereas aldosterone and renin concentrations were within normal limits (renin 10.98 µIU/ml, aldosterone 11.8 ng/dl). Consequently, at a regional hospital, she underwent laparoscopic resection of the right adrenal adenoma, which was histopathologically confirmed to be an adrenocortical adenoma.

Postoperatively, the patient developed symptoms of nausea, fatigue, and headache. She was started on oral prednisone 10 mg daily as replacement therapy, which was gradually reduced to 5 mg daily. Despite surgical intervention, her hypertension remained uncontrolled, with readings between 120–140/70–90 mmHg. Persistent hypokalaemia was also noted (lowest potassium level of 2.86 mmol/L), leading to severe anxiety, depression, declining physical strength, generalized joint pain, and significant weight loss of 15 kg over four months. Subsequently, she was admitted to the Department of Endocrinology and Metabolism in our hospital for further management.

## Diagnostic assessment

Upon admission, her blood pressure was 145/104 mmHg, with a pulse of 78 beats per minute. She measured 154 cm in height, weighed 46 kg, and had a waist circumference of 70 cm. The physical findings included thin skin, extensive striae on the upper arms and thighs, abdominal striae, and postoperative scars, but no pronounced moon facies, buffalo hump, or supraclavicular fat pads. Routine laboratory analysis revealed hypokalaemia (2.86 mmol/l), decreased renin concentrations (<0.50 µIU/ml), elevated aldosterone (19.10 ng/dL), and plasma cortisol concentrations of 109 nmol/L at 8:00 AM and 24.4 nmol/L at midnight. Following correction of the hypokalaemia, plasma aldosterone was not suppressed in the captopril challenge test or the saline infusion test, confirming the diagnosis of primary hyperaldosteronism (Table 1). Considering the recent surgical removal of a cortisol-producing adenoma, the patient was initially treated conservatively with pharmacological therapy. She was prescribed spironolactone 20 mg twice daily for two months, transitioning to terazosin hydrochloride 2 mg daily and nifedipine 30 mg daily for one month, with plans for a comprehensive reassessment upon readmission.

**Table 1:** Diagnostic tests for primary aldosteronism after cortisoloma resection

Time		4 months after cortisoloma resection			7 months after cortisoloma resection	
Hormone	Pre CCT^a^	Post CCT	Pre SIT^b^	Post SIT	Pre CCT	Post CCT
DRC	0.93 uIU/ml	1.41 uIU/ml	<0.5 uIU/ml	<0.5 uIU/ml	1.3 uIU/ml	1.27 uIU/ml
PAC	27 ng/dl	22.9 ng/dl	20.4 ng/dl	15.9 ng/dl	17.3 ng/dl	17.7 ng/dl
ARR	29.03	16.24	-	-	13.31	13.9
K^+^^c^	4.18 mmol/L	4.39 mmol/L	-	4.25 mmol/L	3.85 mmol/L	3.53 mmol/L
PTC	154 mmol/L	74.8 mmol/L	1.19 mmol/L	71.6 mmol/L	193 mmol/L	223 mmol/L

^a^CCT: Captopril Challenge Test

^b^SIT: Saline Infusion Test

^c^potassium levels after potassium supplementation

**Fig. 2:**
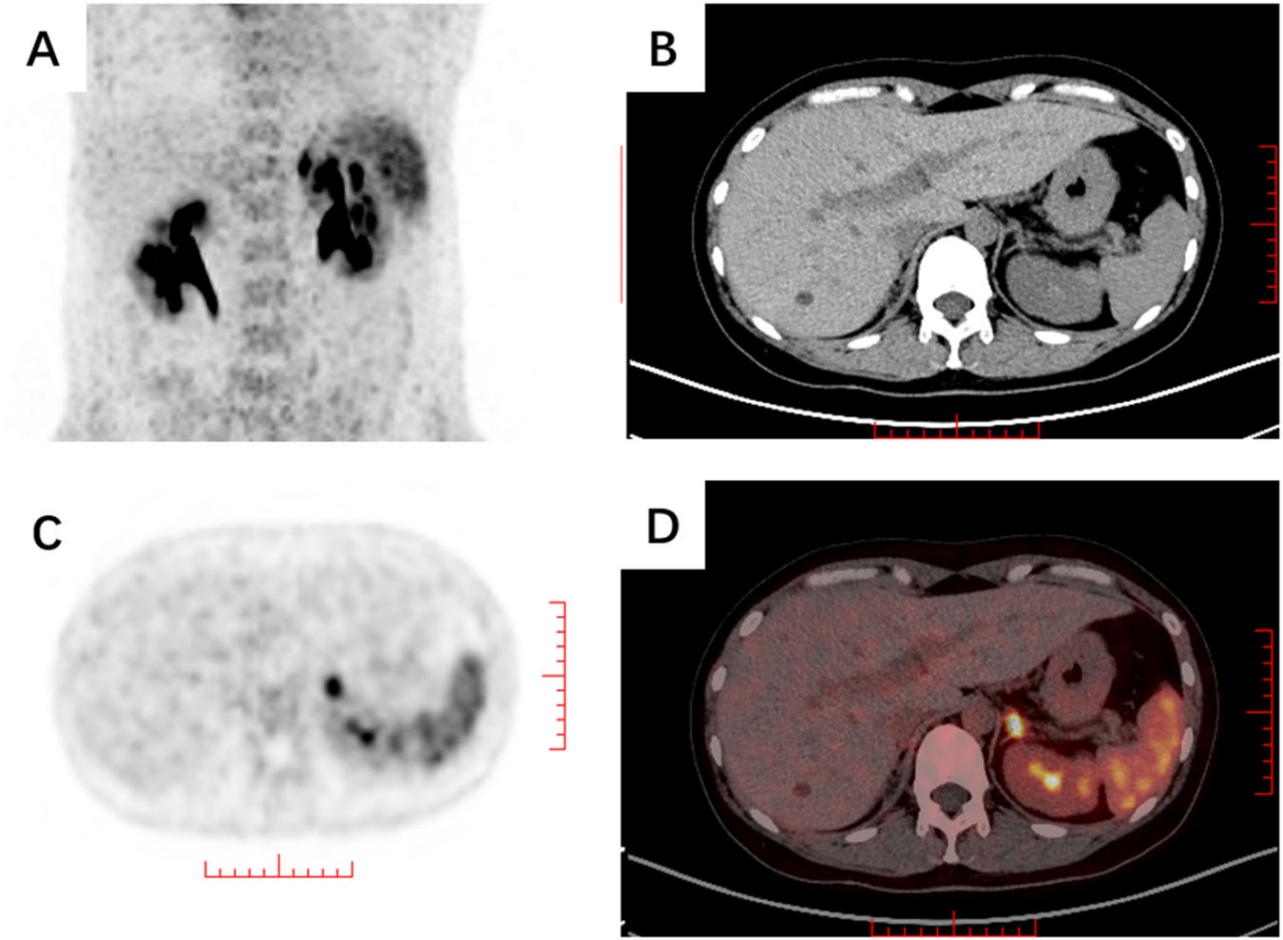
CXCR4-targeted PET/CT nuclear imaging results. **A** PET maximum intensity projection shows increased metabolic activity near the medial limb of the left adrenal body. **B**–**D** Unenhanced and contrast-enhanced CT and fused images display a uniform density left adrenal nodule with increased metabolism, SUVmax 8.75

The follow-up results indicated a direct renin concentration of 1.49 µIU/ml, a plasma aldosterone concentration of 22 ng/dl, and an aldosterone-renin ratio (ARR) of 14.77 ng/dl/µIU/ml, with positive outcomes on related functional tests (Table 1). After a low-dose dexamethasone suppression test, the morning cortisol concentration was 12.4 nmol/L. Whole-blood genetic testing revealed no abnormalities, ruling out familial primary hyperaldosteronism. The diagnosis of primary aldosteronism was confirmed. To further ascertain whether the left-side adrenal mass was an aldosteronoma, we utilized 68 Ga-Pentixafor PET/CT imaging targeting the C-X-C chemokine receptor type 4 (CXCR4), which indicated that the maximum standard uptake value (SUVmax) of the left adrenal nodule was 8.75, whereas it was 2.7 in the normal left adrenal gland, with an average SUV of 2.34, a lesion to normal adrenal ratio (LAR) of 3.74, and a lesion-to-liver ratio (LLR) of 8.83. The right adrenal gland showed no definite lesions, suggesting a high likelihood of an aldosteronoma on the left side (Fig. 2).

## Treatment

Considering that the patient had undergone the removal of a cortisol-producing adenoma several months earlier, after discussion with the patient, we opted to proceed with surgical removal of the left aldosteronoma. To assess adrenal cortical function recovery, an ACTH stimulation test was conducted preoperatively. The cortisol peak reached 534 nmol/L at ninety minutes after the administration of ACTH, indicating that the patient’s adrenal reserve function was essentially normal. The left adrenal tumor was then surgically removed. Hydrocortisone was administered immediately after the operation to prevent adrenal crisis. On postoperative day one, the patient’s blood pressure and potassium levels normalized, and all antihypertensive medications were discontinued.

## Outcome and follow-up

**Fig. 3:**
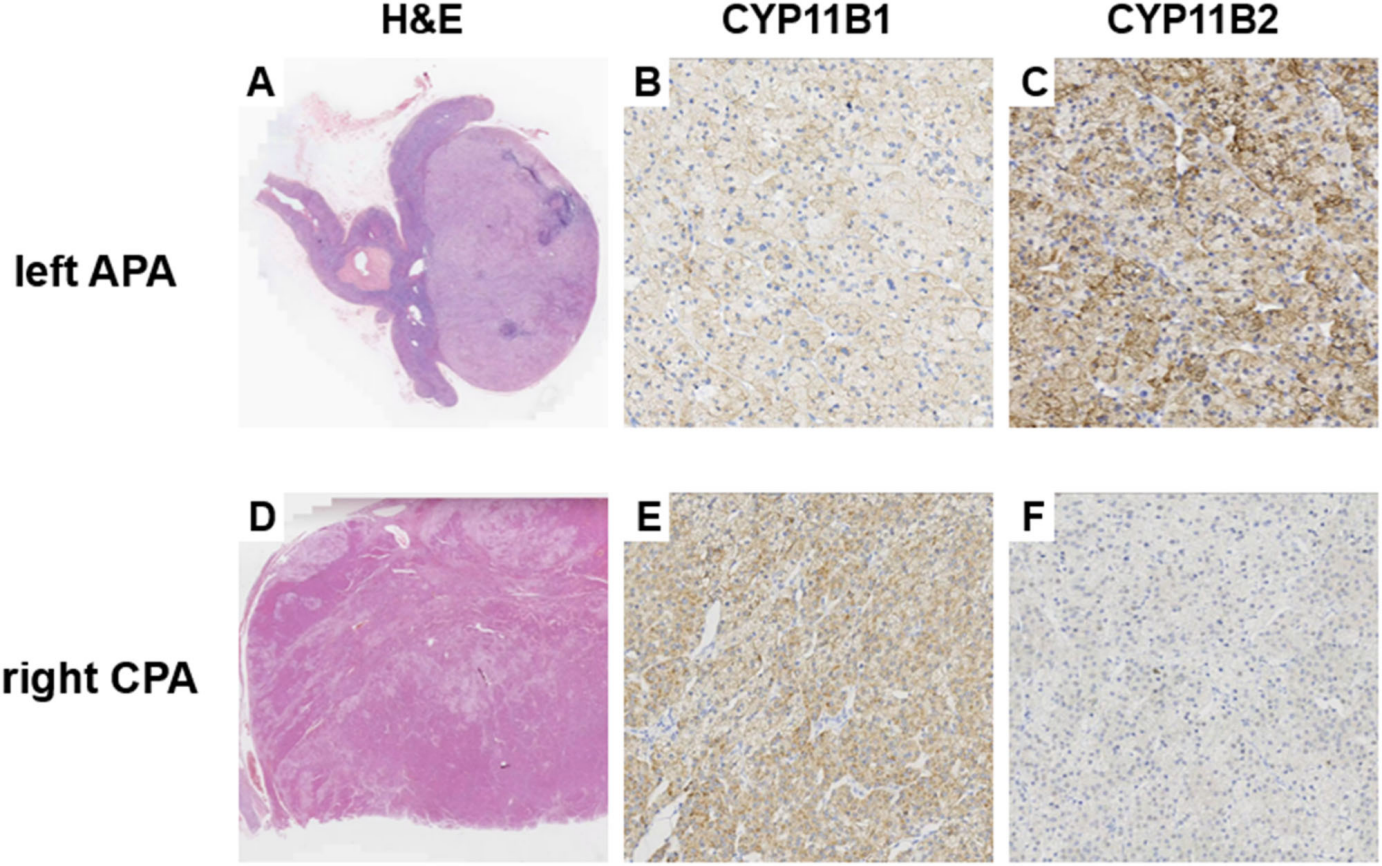
Histological and immunohistochemical examination results: **A** Left adrenal gland, predominantly composed of eosinophilic compact cells (H&E staining). **B** Immunohistochemical staining (x200) for CYP11B1 in the left adrenal gland, showing CYP11B1 (−). **C** Immunohistochemical staining (x200) for CYP11B2 in the left adrenal gland, showing CYP11B2 (+). **D** Right adrenal gland, primarily composed of clear cells and compact cells with the former being predominant (H&E staining). **E** Immunohistochemical staining (x200) for CYP11B1 in the right adrenal gland, showing CYP11B1 (+). **F** Immunohistochemical staining (x200) for CYP11B2 in the right adrenal gland, showing CYP11B2 (−)

**Fig. 4:**
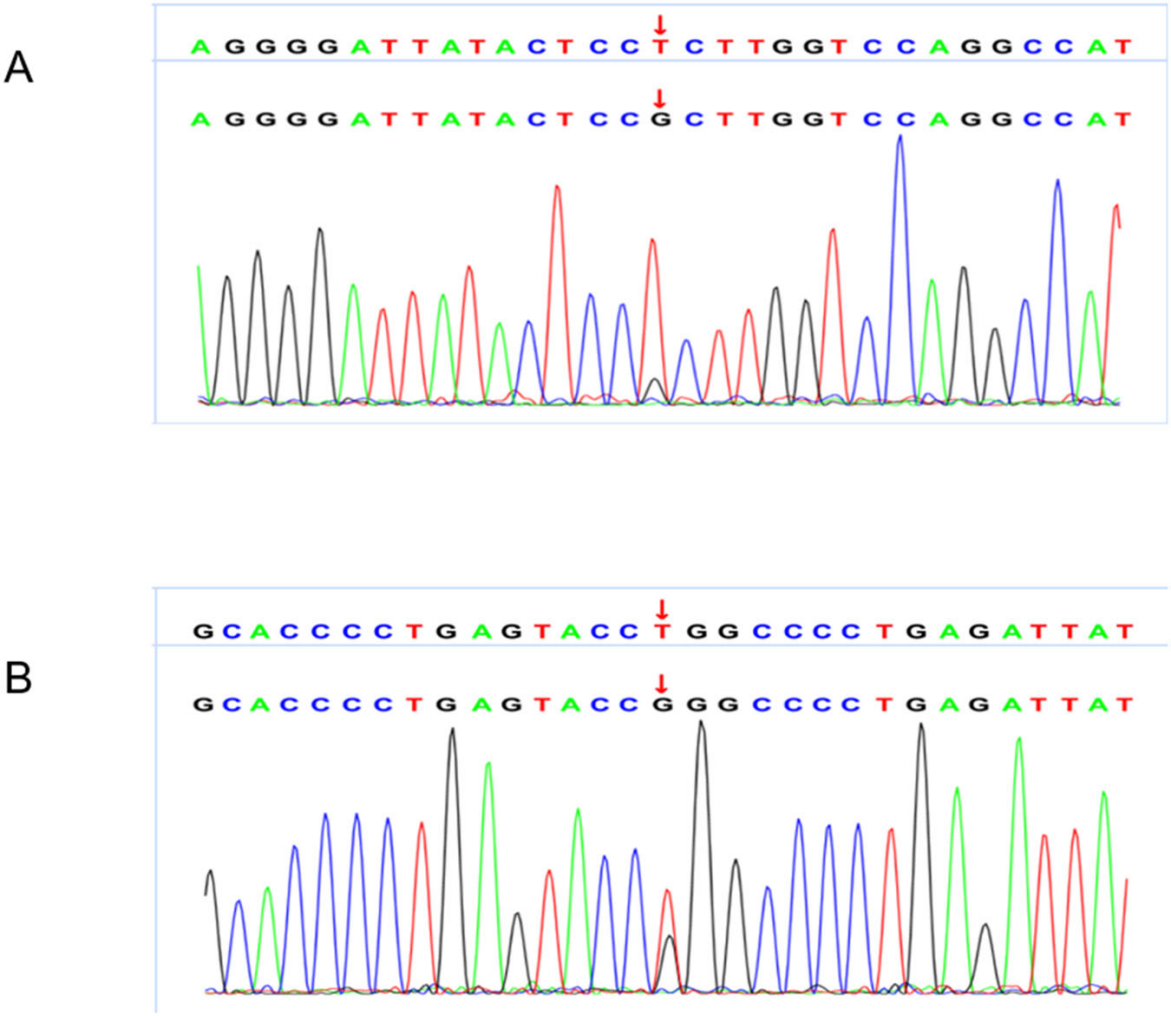
Sequencing chromatograms showing somatic mutations identified in bilateral adenomas. **A** Identification of a heterozygous KCNJ5 c.503T > G somatic mutation in the left adrenal adenoma. **B** Identification of a PRKACA c.617(exon7)T > G somatic mutation in the right adrenal adenoma

Pathology analysis revealed a 1.3 × 1 × 1 cm golden-colored adenoma in the left adrenal gland. Further histopathological examination with haematoxylin and eosin (H&E) staining revealed that the left adrenal cortical adenoma predominantly consisted of clear cells, whereas the right adrenal cortical adenoma was characterized by a mixture of eosinophilic compact cells and clear cells. Immunohistochemical analysis confirmed distinct hormone synthesis profiles: the left adenoma diffusely expressed CYP11B2 without CYP11B1 expression, which was indicative of an aldosterone-producing adenoma, while the right adenoma exhibited diffuse expression of CYP11B1 and the absence of CYP11B2, which was consistent with a cortisol-secreting phenotype (Fig. 3). Whole exome sequencing of both tumors and peripheral blood identified pathogenic mutations, with the cortisol-producing adenoma on the right harboring a PRKACA c.617(exon7)T > G (p.L206R) mutation and the aldosterone-producing adenoma on the left showing a somatic heterozygous missense mutation in KCNJ5 c.503(exon2)T > G (p.L168R) (Fig. 4).

One month postoperatively, the patient presented with elevated renin concentrations (129.2 µIU/ml) and normal sodium levels (134.4 mmol/L); her cortisol level was 94.1 nmol/L, and her ACTH level was 146.1 ng/L. Symptoms of dizziness, fatigue, and palpitations were reported, which improved with the addition of 0.05 mg of fludrocortisone daily to the ongoing hydrocortisone therapy. At the two-month follow-up, the patient’s sodium level had adjusted to 136.1 mmol/L, her ACTH level had increased to 111.9 ng/L, and her blood pressure had normalized, but she continued to experience occasional dizziness and headaches. Fludrocortisone was increased to 0.075 mg daily, and hydrocortisone was adjusted to 5 mg every other day, in accordance with dietary recommendations for increased salt and fluid intake. After two months of following this regimen, the patient’s electrolytes normalized without further complaints, leading to a reduction in fludrocortisone to 0.025 mg daily while maintaining the hydrocortisone dosage. Three months later, the electrolytes remained normal, the morning cortisol level was 304 nmol/l, and the ACTH level was 69.54 ng/dl, with a normal RAAS, prompting discontinuation of the medications (Table 2). The patient reported occasional aversion to oily foods and dyspepsia, with no change in her weight. A repeat ACTH stimulation test indicated a peak cortisol concentration of 621 nmol/l at 120 min post-administration, confirming full recovery of adrenal function.

**Table 2:** Follow-up after adrenalectomy for bilateral adrenal adenomas

Hormone Tested	1 month after surgery	3 month after surgery	6 month after surgery	9 month after surgery
DRC	129.2 uIU/ml	70.53 uIU/ml	130.6 uIU/ml	46.4 uIU/ml
PAC	10.5 ng/dl	8.26 ng/dl	10.4 ng/dl	8.52 ng/dl
ARR	0.08	0.12	0.08	0.19
PTC	94.1 mmol/L	203 mmol/L	347 mmol/L	304 mmol/L
ACTH	146.1 ng/l	111.9 ng/l	85.61 ng/l	69.54 ng/l
K^+^	4.46 mmol/L	4.43 mmol/L	4.89 mmol/L	4.39 mmol/L

## Discussion

Hypertensive disorders affect approximately 5–10% of pregnancies and are a leading cause of maternal, foetal, and neonatal morbidity and mortality [1]. The vast majority of these cases are caused by primary hypertension, with a prevalence of 1.52%. Although secondary hypertension accounts for only 0.24% of cases [2], it is associated with a significantly increased risk of adverse outcomes for both mothers and foetuses. The possibility of secondary hypertension should be considered, especially when it is accompanied by hypokalaemia. Primary aldosteronism is the most common cause of secondary hypertension, and aldosterone/cortisol co-secreting tumours represent a special subtype of PA, accounting for 5.31–21% of PA cases [3]. These tumours are characterized by the abnormal secretion of both aldosterone and cortisol. Research on the incidence and prevalence of PA and Cushing’s syndrome (CS) during pregnancy is extremely limited, and the literature consists of single case reports and studies involving a small number of patients. Currently, there are no reports of pregnancy complicated by aldosterone/cortisol co-secreting tumours.

Normal pregnancy is often characterized by elevated cortisol levels. According to the latest consensus [4], the course of normal pregnancy is referred to as nontumorous hypercortisolism (formerly known as pseudo-Cushing’s syndrome). During this period, owing to the stimulation of the pituitary and placenta by corticotropin-releasing hormone (CRH), the synthesis and secretion of ACTH increase, promoting adrenal hypertrophy and increased cortisol production [5]. In turn, high cortisol levels stimulate the placenta to synthesize and release more CRH, creating a positive feedback loop that maintains elevated cortisol levels [6]. This makes it extremely challenging to confirm the presence of CS through laboratory tests during pregnancy. In normal pregnant women, the false-positive rate of the low-dose dexamethasone suppression test (DST) is as high as 80% [7]. Although elevated midnight plasma and salivary cortisol levels are potential diagnostic markers, their diagnostic thresholds have yet to be established during pregnancy [8]. Moreover, overlapping clinical features between CS pregnancies and normal pregnancies, such as weight gain, abdominal striae, and peripheral edema, often lead to delayed diagnosis in practice. Currently, there is no consensus on the treatment of such patients. In this case, after antihypertensive and potassium supplementation treatments proved ineffective, the obstetrician opted for a caesarean section after adequate preoperative preparation. Fifty days postpartum, the persistence of hypertension and hypokalaemia suggested the presence of a pathological condition unrelated to pregnancy. At that time, abdominal CT revealed bilateral adrenal adenomas, and elevated cortisol levels with suppressed ACTH helped confirm the diagnosis of ACTH-independent Cushing’s syndrome.

Due to the limitations in diagnostic capabilities at the local hospital, the surgeon chose to remove the larger right adrenal tumor without fully characterizing the functional status of the bilateral adrenal adenomas, a decision that aligns with some current treatment recommendations [9]. Postoperative pathological examination and subsequent signs of overt adrenal insufficiency provided indirect evidence that the removed tumor was a hyperfunctioning cortisol-secreting adenoma. However, the patient’s persistent hypertension after surgery suggested the presence of additional unrecognized pathologies, whereas her continuous hypokalaemia and the remaining adrenal adenoma suggested a diagnosis of primary aldosteronism. Although the ARR was negative during the first screening, this may have been due to the overall upregulation of the RAAS during pregnancy. Oestrogen produced by the placenta increases the hepatic synthesis of angiotensinogen [10], whereas renal oestrogen stimulation and extrarenal synthesis in the ovaries and maternal decidua lead to a significant increase in the plasma renin concentration and activity [11]. Increased renin activity promotes elevated angiotensin II levels, which in turn stimulates aldosterone production in the zona glomerulosa. After delivery, the sharp decline in progesterone and estrogen levels following placental expulsion leads to a decrease in the ARR. The relatively high and proportionate increase in renin activity may further reduce the ARR, potentially increasing the likelihood of false-negative results. Furthermore, abnormal increases in cortisol levels can also elevate angiotensinogen levels [12], and the use of nifedipine can make test results unreliable. After adjusting the medication, we reinitiated the diagnostic process for primary aldosteronism; during this time, the patient exhibited a significant decrease in renin and an elevated ARR, and both captopril and saline infusion tests confirmed autonomous aldosterone secretion, establishing a diagnosis of primary hyperaldosteronism.

Differentiating the subtype of primary aldosteronism and the secretory function of adrenal lesions is crucial for managing primary aldosteronism. Given our patient’s youth and autonomous cortisol hypersecretion, genetic disorders such as familial hyperaldosteronism or hereditary Cushing’s syndrome had to be considered, but whole-exome sequencing of peripheral blood did not reveal any significant pathogenic gene defects. We then focused on determining the subtype of primary aldosteronism. Although adrenal venous sampling (AVS) is universally regarded as the “gold” standard for distinguishing between unilateral and bilateral secretion in primary aldosteronism patients, its interpretation becomes challenging in patients with concomitant high cortisol secretion. In this case, although the factor causing autonomous cortisol secretion was surgically removed, postoperative adrenal insufficiency and glucocorticoid replacement therapy could still interfere with the serum cortisol concentrations in the inferior vena cava and adrenal veins. Additionally, AVS is an invasive procedure and the patient had undergone two invasive abdominal surgeries in a short period, namely, a caesarean section and right adrenal tumor resection, which significantly increased the risk of AVS failure and complications. Thus, AVS was deemed unsuitable for this case.

Recent studies have verified that 68Ga-Pentixafor PET/CT imaging, which targets the CXCR4 receptor, demonstrates substantial concordance with AVS in differentiating the subtypes of primary aldosteronism [12]. For nodules larger than 1 cm in PA patients, when the SUVmax is greater than 7.3, the specificity of CXCR4 imaging for identifying APA is 100% [13]. Therefore, we opted for CXCR4 PET/CT imaging to ascertain the functional status of the adrenal lesion. The results revealed that the left adrenal adenoma had a maximum SUV of 8.75, which was significantly greater than that of the surrounding adrenal tissue (a maximum SUV of 2.7 and an average SUV of 2.34), confirming that the adenoma was an aldosteronoma. The patient was thus diagnosed as having a rare case of A/CPA with a right adenoma secreting cortisol and a left adenoma secreting aldosterone. However, notably, individual studies have reported increased uptake of CXCR4-targeted 68 Ga-PentixaFor in cortisol-producing adrenal adenomas. Therefore, this technique needs to be combined with hormone assays and functional tests to more accurately differentiate functional adrenal adenomas[14].

For the treatment of unilateral aldosteronoma, both national and international guidelines recommend complete adrenal resection, but there is currently no consensus on the surgical approach for A/CPA. The average incidence of postoperative adrenal insufficiency in Cushing’s syndrome patients is as high as 99.7% [15], and avoiding permanent adrenal insufficiency is a crucial factor in determining the surgical approach. Since the patient had already undergone resection of the right adrenal cortical adenoma and experienced adrenal insufficiency, further resection of the left adrenal gland would have significantly increased the risk of permanent adrenal insufficiency. Given that CT and CXCR4 imaging revealed no abnormal changes beyond the tumors, we opted to remove the left adrenal tumor while preserving the remaining adrenal tissue. The surgery was scheduled nine months after the first operation, as ACTH stimulation tests indicated nearly normal adrenal function. This treatment approach proved effective over a one-year follow-up. After the operation, the patient’s hypertension and hypokalaemia were completely relieved. However, adrenal insufficiency appeared as predicted, for which we administered glucocorticoid and mineralocorticoid replacement therapy, later adjusting the dosage on the basis of follow-up findings and finally discontinuing the treatment after nine months. Biochemical tests and ACTH stimulation tests confirmed normal adrenal cortical function, and the patient felt well, validating our treatment approach. Thus, the choice of surgical approach for patients with bilateral adrenal adenomas that secrete aldosterone and cortisol independently should be made while considering the specific location and secretion status of the adrenal tumors, as well as the patients’ individual circumstances.

Given the absence of germline mutations in the peripheral blood, we performed immunohistochemical and genetic analyses of the bilateral tumors. In the left adrenal adenoma, a classical somatic missense mutation in KCNJ5, c.503(exon2)T > G (p.L168R), was detected, and immunohistochemistry revealed predominant expression of CYP11B2, indicating that it was the source of autonomous aldosterone production. Research has confirmed that the p.L168R mutation of the KCNJ gene leads to depolarization of the ZG cell membrane, allowing an influx of extracellular calcium ions, which activates aldosterone synthesis and secretion [16]. On the right side, the adenoma harbored a PRKACA c.617(exon7)T > G (p.L206R) mutation with immunohistochemistry confirming strong CYP11B1 expression. This p.L206R mutation activates PKA independently of cAMP, disrupting normal cortisol regulation and leading to autonomous glucocorticoid secretion and Cushing’s syndrome development [17]. However, whether pregnancy is a potential predisposing factor for the simultaneous occurrence of two adenomas independently secreting different hormones in a single patient, or if this case is merely the result of coincidence, requires further investigation with a larger sample size.

## Results

During pregnancy, complex endocrine changes can easily obscure underlying pathological conditions, especially in cases where Cushing’s syndrome and primary aldosteronism coexist, making timely diagnosis extremely difficult and often leading to delays. For patients with refractory hypertension and hypokalemia during pregnancy, the possibility of endocrine hypertension should be highly suspected, and early interdisciplinary approaches are crucial for optimizing maternal and fetal outcomes. For young patients with endocrine hypertension, it is essential to first rule out potential genetic causes. In patients with primary aldosteronism coexisting with Cushing’s syndrome or receiving exogenous glucocorticoid supplementation, CXCR4 imaging is more suitable than AVS for determining lateralization. This noninvasive technique not only clarifies the subtype of primary aldosteronism but also provides comprehensive diagnostic information, offering important guidance for treatment planning and management decisions. With respect to surgical options, for patients with bilateral adrenal adenomas independently secreting aldosterone and cortisol, bilateral tumor resection may be the most appropriate surgical strategy, as it minimizes the risk of permanent adrenal insufficiency postoperatively. Perioperative glucocorticoid therapy, along with monitoring and supplementation for mineralocorticoid deficiency, can prevent adrenal crises and help gradually restore adrenal cortical function through careful dose adjustments. Our case presents an extremely rare instance of pregnancy complicated by bilateral adrenal adenomas independently secreting cortisol and aldosterone, providing a reference for the diagnosis and treatment of endogenous Cushing’s syndrome and primary aldosteronism during pregnancy. These condition were further confirmed to be caused by independent somatic mutations in PRKACA and KCNJ5. However, whether pregnancy is a potential predisposing factor for bilateral adrenal somatic mutations in the same patient requires further research with larger sample sizes to elucidate the underlying mechanisms.

## Learning points

- The complex endocrine changes during pregnancy can mask underlying pathological conditions. In cases of refractory hypertension and hypokalaemia during pregnancy, endocrine hypertension should be highly suspected. Early interdisciplinary approaches are crucial for improving maternal and foetal outcomes.
- A/CPA is relatively common and warrants clinical vigilance, particularly during the diagnosis of primary aldosteronism, where excessive cortisol levels and physiological changes such as pregnancy may obscure accurate mineralocorticoid assessment.
- CXCR4 imaging, which is preferable to AVS because of its noninvasiveness, can be used to effectively delineate primary aldosteronism subtypes and inform treatment planning.
- In patients with bilateral adrenal adenomas that secrete both aldosterone and cortisol, bilateral tumor resection is advised to avoid permanent adrenal insufficiency. Perioperative management with glucocorticoids and mineralocorticoids prevents adrenal crises and facilitates adrenal function recovery.
- Our case, which uniquely documented bilateral adenomas caused by distinct mutations in KCNJ5 and PRKACA, highlights the need for further large-scale studies to better understand and treat this condition.
